# Rethinking rurality: using hospital referral regions to investigate rural-urban health outcomes

**DOI:** 10.1186/s12913-022-08649-0

**Published:** 2022-11-03

**Authors:** Lucy Skinner, Sandra Wong, Carrie Colla

**Affiliations:** 1 Geisel School of Medicine at Dartmouth, Hanover, NH US; 2 Department of Surgery, Geisel School of Medicine at Dartmouth, Hanover, Dartmouth-Hitchcock, NH US; 3 The Congressional Budget Office, Washington, DC US; 4 The Dartmouth Institute for Health Policy and Clinical Practice, Geisel School of Medicine at Dartmouth, Hanover, NH USA

**Keywords:** Geography, Health disparities, Hospitals, Social determinants of health

## Abstract

**Background:**

Rural residents in the United States face disproportionately poorer health outcomes compared to urban residents. This study aims to establish a continuous rural-urban measure for the 306 hospital referral regions (HRRs) in the U.S. and to investigate the relationship between the proportion of rural population served in each HRR and health outcomes, healthcare spending and utilization, and access to and quality of primary care.

**Methods:**

Cross-sectional analysis using data from The Dartmouth Atlas and the U.S. Census. The sample is limited to fee-for-service Medicare beneficiaries aged 65–99 years and living during 2015. The primary outcomes were measured at the HRR-level: mortality rates, Medicare reimbursements, percent Medicare enrollees who have at least one visit to a primary care physician, diabetic hemoglobin A1c testing rates, and mammography rates. We calculate a population-weighted rural proportion and population-weighted area deprivation index (ADI) for each HRR by aggregating zip-code level data.

**Results:**

The most rural quartile of HRRs had significantly greater mean mortality rate of 4.50%, compared to 3.95% in most urban quartile of HRRs (p < 0.001). Increasing rural proportion was associated with decreasing price-adjusted Medicare reimbursements. In the multivariate, linear regression model, increasing area deprivation (ADI) was associated with increasing rates of mortality and greater utilization.

**Conclusion:**

Disparities in rural mortality are driven by sociodemographic disadvantage, rather than the quality of care provided at hospitals serving rural areas. After accounting for sociodemographic disadvantage, rural areas achieve similar quality of primary care in measured domains at an overall lower cost.

**Supplementary Information:**

The online version contains supplementary material available at 10.1186/s12913-022-08649-0.

## Introduction

In the United States, people living in rural areas face disproportionately poorer health outcomes compared to those living in urban areas [1–3]. Individuals in rural areas have higher rates of morbidity, mortality, smoking, obesity, and opiate-use disorder compared to their urban counterparts [4]. Differences in socioeconomic and demographic factors such as income, education, employment, race/ethnicity distribution, primary care physician supply, and health insurance status across rural and urban areas have been used to explain the disparity in rural health outcomes [1, 5].

Mortality rates in rural compared with urban areas differ across states, with the highest rural mortality disparities in Virginia, Florida, and California, while Wyoming, Colorado, and Montana have lower mortality rates in rural compared with urban areas [5]. These finding suggest that there are regional factors that impact differences in mortality between rural and urban dwellers. Prior research in rural-urban health disparities have used data at the state, county, or national level, but these units of analysis may not capture where rural populations are receiving healthcare services or account for geographic patterns of healthcare utilization [6]. For example, people within the same state may receive major surgical procedures in their neighboring state or county, or there may be two major centers within one state that provide varying quality of care and serve different populations. Using a geographic-based measure of where individuals are accessing care incorporates the nuances of quality of healthcare received at a given hospital into the analysis of rural-urban disparities and provides a better understanding of drivers of the established variation in rural-urban disparities across regions of the U.S.

Hospital Referral Regions (HRRs) are 306 geographic areas created by the Dartmouth Atlas project to characterize where Medicare recipients are admitted for tertiary care. HRRs provide a more accurate measure of where the population within a certain region access inpatient healthcare services compared to other units of analyses based on county or state boundaries [7]. HRRs have not previously been given a rural-urban designation. This study has two aims: first, to establish a rural-urban measure for the 306 HRRs based on the rural/urban designations of populations they serve, and second, to use the rural-urban HRR measure to analyze health disparities, including an investigation into whether mortality differences are correlated with differences across regions in price-adjusted Medicare reimbursements, as well as quality of and access to primary care. By conducting rural-urban comparisons using HRRs, this study aims to better understand the drivers of previously established rural health disparities and to investigate whether health outcomes in rural areas differ due to population-level characteristics such as income, education, and employment, or the quality of care available to rural populations [7].

## Methods

### Study population

This study is limited to fee-for-service U.S. Medicare beneficiaries aged 65–99 years. Beneficiaries who die during the study year are excluded.

### Hospital Referral Regions

Hospital referral regions (HRRs) represent the healthcare market for a tertiary hospital. To designate HRRs, the Dartmouth Atlas project assigned 5-digit zip codes to smaller units called Hospital Service Areas (HSAs), based on the hospital where the plurality of Medicare beneficiaries who reside within that zip code are hospitalized. The Dartmouth Atlas then aggregates HSAs into larger-area HRRs based on where the Medicare enrollees within that HSA access major cardiovascular procedures or neurosurgery [8]. There are 3,436 HSAs and 306 HRRs in the U.S. Medicare Advantage are only included in mortality outcome measures, and are not included in Medicare reimbursement measures as Medicare Advantage is a Health Maintenance Organization (HMO) and not fee-for-service so cannot be included in the analysis. We used the MABLE-14 crosswalk geographic database to create a custom data set with zip codes, HRR, and total populations of each zip code [9].

### Area Deprivation Index

Area deprivation index (ADI) is a measure of socioeconomic disadvantage derived from the American Community Survey assigned as a national ranking from 1 to 100, where 100 is most deprived, based on a cumulative measure of income, education, employment, and housing quality in a given census block-group/neighborhood. We use the national rankings of ADI assigned at the zip code level. Higher national rankings of ADI correspond to greater levels of disadvantage [10].

### Outcome variables

Medicare reimbursements, mortality rates, and primary care access and quality data are publicly available from the Dartmouth Atlas Project website [11]. Price-adjusted Medicare reimbursements are a measure of health care utilization, and were calculated from a 20% Medicare sample using diagnostic-related group (DRG) pricing and adjusted based on variation in Medicare reimbursement by region from 2017 [6]. Mortality rates were measured among 2017 Medicare beneficiaries. Primary care access was calculated as a rate with the number of Medicare beneficiaries with at least 1 primary care visit (in 2015) divided by the total number of beneficiaries in the given region from the 20% Medicare sample. Quality of primary care was determined by proportion of diabetic enrollees who receive a hemoglobin A1c test and proportion of eligible women who receive mammograms [12].

### Data aggregation

Rural-urban commuting area (RUCA) codes assign a value 1–10 to each zip code (1 representing most urban, 10 most rural) and are determined by measures of population density, urbanization, and daily commuting from the 2010 decennial census data from the United States Department of Agriculture Economic Research Service [13]. We merged the RUCA-zip code, HRR-zip code, and ADI-zip code data sets based on zip code to create a single dataset with an identified HRR, ADI, total population, and RUCA code for each zip code. We collapsed the dataset on HRR, creating a total population residing within each RUCA code in the HRR, the sum total population of the HRR, and a population-weighted measure of ADI equal to the zip code ADI multiplied by the zip code population divided by total population of the HRR.

### Calculating rural proportion

For each HRR, we calculated a proportion value for RUCA codes 1–10 based on the proportion of the population of that HRR that lives within each RUCA code. We used the Health Resources Services Administration (HRSA) definition of rural as RUCA codes 4–10 and urban as RUCA codes 1–3 and created a population-weighted measure of rural and urban based on the total population in the rural and urban RUCA code groupings.

### Analysis

We examined the linear correlation between rural proportion and ADI using Spearman’s rank correlation coefficient. We then compared unadjusted summary measures of our outcomes in the quartile with the most rural HRRs to the quartile with the most urban (least rural) HRRs, assessing significant differences in groups using a two-sample t-test. We performed univariate and multivariate linear regression analyses to investigate the relationship between rural proportion and our outcomes measures. The data met the assumptions for t-test and multivariable linear regression. The fully-adjusted linear regression model included rural proportion and ADI. We determined significance at the p = 0.05 level. Data was analyzed using Stata version 16.0. (Statacorps, 2017) [14].

## Results

### Rural proportion measure and area deprivation index

**Fig. 1:**
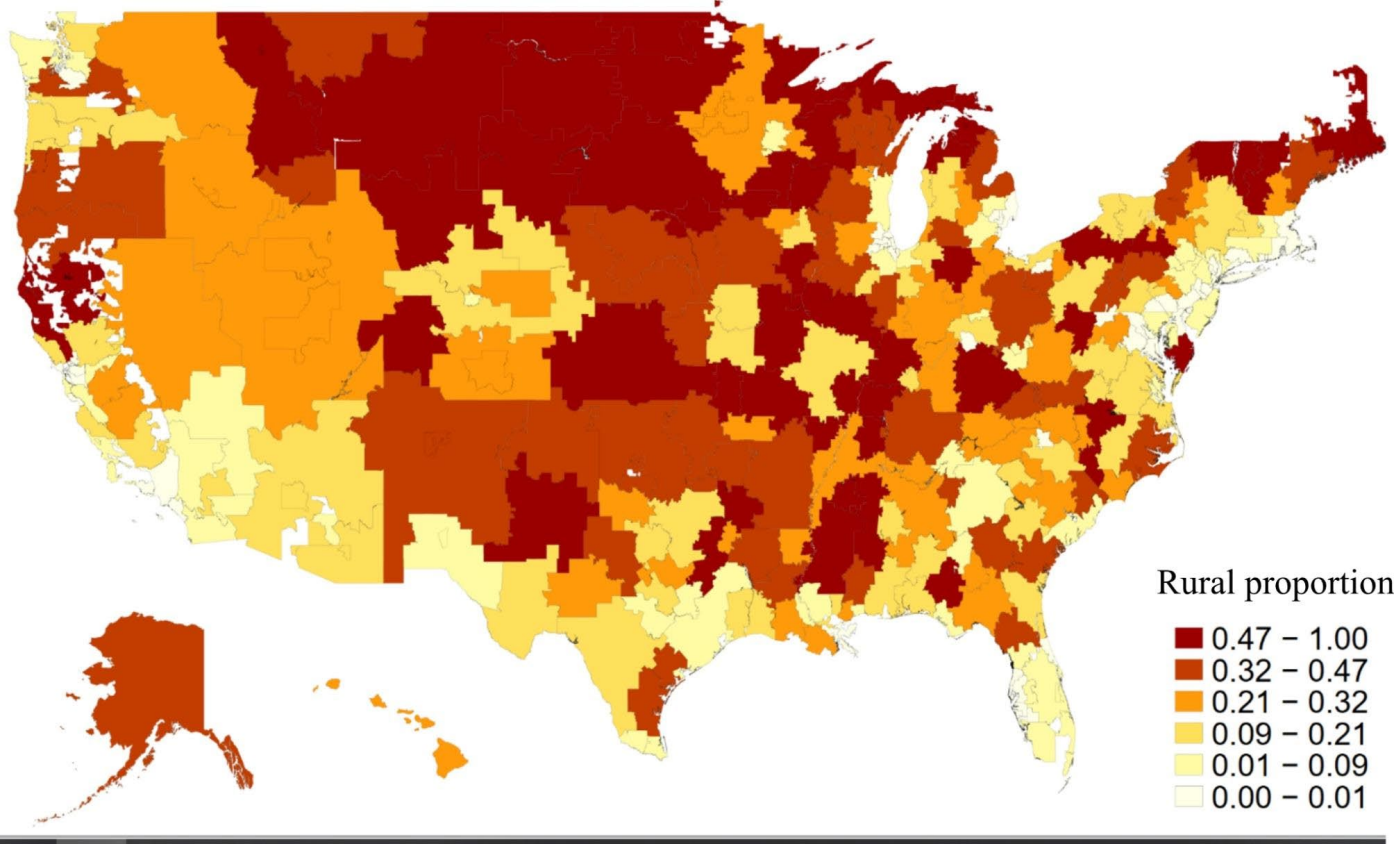
Proportion of population that is designated as rural within each of the 306 Hospital Referral Regions in the U.S. Figure 1 was created using a Stata program created by Dr. Michael Stepner [26]

**Fig. 2:**
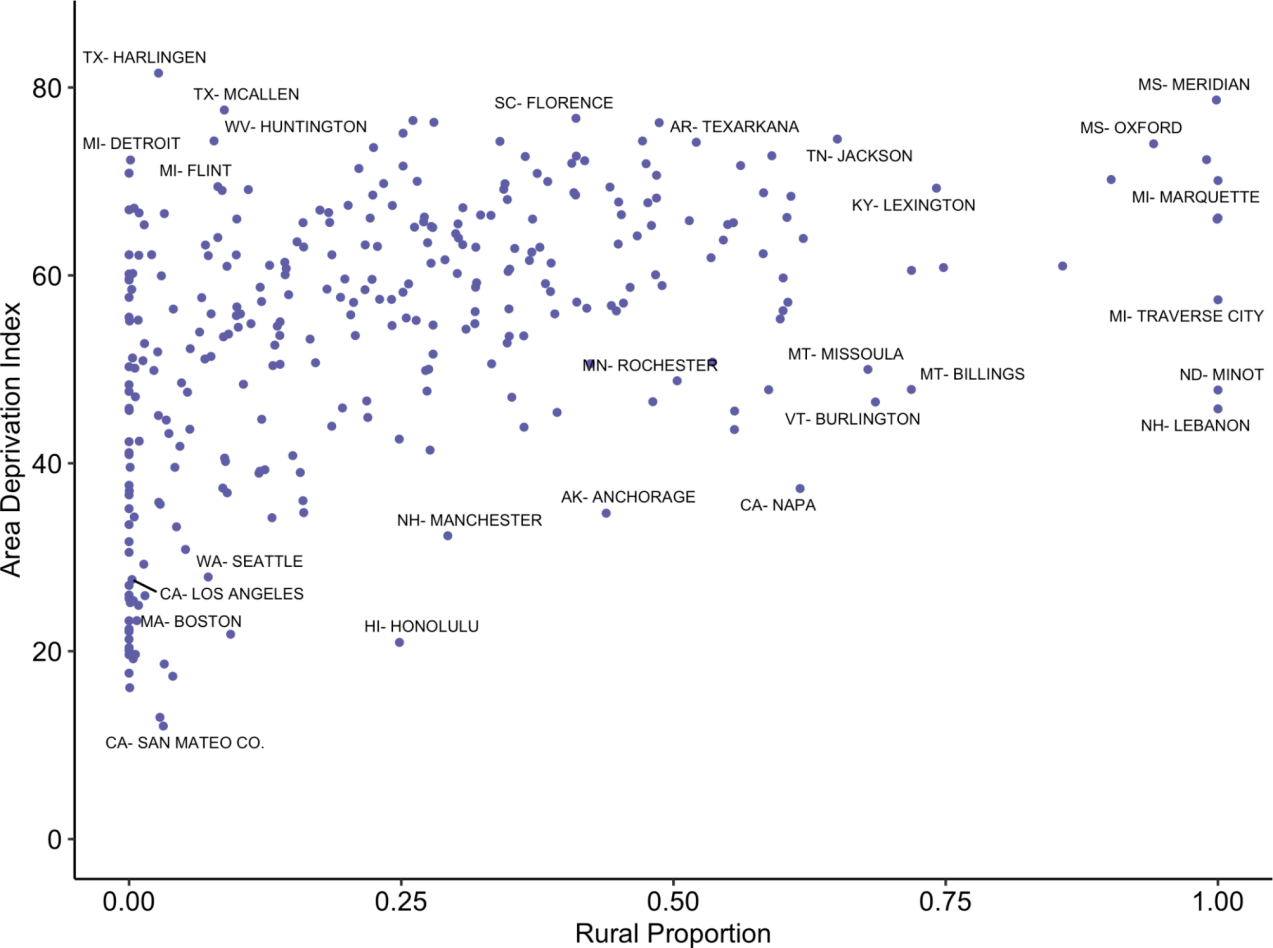
Scatterplot of rural proportion of population and area deprivation index for each of the 306 HRRs in the U.S. Spearman’s ρ = 0.49 (p < 0.001)

The 306 U.S. HRRs were assigned a measure of proportion rurality ranging from 0 to 1. Figure 1 shows the proportion rurality measure for each HRR represented by color density. The median proportion rurality was 0.207. Population-weighted ADI for each HRR ranges from 12.0 to 81.5, with a median of 57.1. The relationship between rural proportion and ADI is shown in Fig. 2, Spearman’s ρ = 0.49 (p < 0.001).

### Unadjusted results

The most rural quartile HRRs had significantly greater mean population-weighted ADI of 61.7 (95%CI: 59.5, 64.0) compared with 41.6 (95% CI: 37.7, 45.4) in the most urban quartile and the most rural quartile had significantly greater age, sex, race adjusted percent mortality of 4.50% (95%CI: 4.40%, 4.59%) compared to 3.95% (95% CI: 3.84%, 4.06%) in the most urban quartile HRRs. The rural quartile HRRs had lower total and price-adjusted Medicare reimbursements of $9,506 (95%CI: $9,324, $9,969) and $10,049 (95%CI: $9,777, $10,322), respectively, compared to the total and price adjusted Medicare reimbursements in the most urban quartile HRRs of $11,180 (95% CI: $10,895, $11,466) and $10,694 (95% CI: $10,427, $10,962), respectively. In the measures of primary care access and quality, the most rural quartile of HRRs had a greater percentage of Medicare enrollees who have at least one primary care visit, with 80.8% (95% CI: 79.9%, 81.7%) compared to 77.4% (95% CI: 76.2%, 78.6%) for the most urban quartile. The other measures of primary care quality, the percent of diabetic enrollees receiving hemoglobin A1c tests and the percent of eligible female enrollees receiving mammograms, did not differ significantly between the most rural and urban HRRs (Table 1).

**Table 1:** Mean values (95% CI) for the highest quartile rural (N = 77) and highest quartile urban (N = 77) hospital referral regions

	Rural *Top quartile ‘most rural’*	Urban *Top quartile ‘most urban’*	p-value*
Area Deprivation Index (ADI)	61.7 (59.5, 64.0)	41.6 (37.7, 45.4)	< 0.001
Total mortality ^1^	4.50% (4.40%, 4.59%)	3.95% (3.84%, 4.06%)	< 0.001
Total Medicare reimbursement ^2^	$9,506 ($9,324, $9,969)	$11,180 ($10,895, $11,466)	< 0.001
Price-adjusted Medicare reimbursement ^2^	$10,049 ($9,777, $10,322)	$10,694 ($10,427, $10,962)	0.001
Annual primary care visit ^3^	80.8% (79.9%, 81.7%)	77.4% (76.2%, 78.6%)	< 0.001
Diabetic enrollees who receive hemoglobin A1c test ^4^	85.8% (85.0%, 86.7%)	85.6% (85.0%, 86.2%)	0.676
Percent of eligible female enrollees who receive mammogram ^5^	62.9% (61.7%, 64.2%)	64.5% (63.0%, 65.4%)	0.179

^1^Percent of deaths among Medicare enrollees adjusted for age, sex, and race

^2^Total annual reimbursements per Medicare enrollee (parts A & B)

^3^Percent of Medicare enrollees who have at least 1 visit to a primary care clinician

^4^Average annual percent of diabetic Medicare enrollees age 65–75 who receive hemoglobin A1c test

^5^Average percent of female Medicare enrollees age 67–69 having at least one mammogram over a two-year period

^*^T-tests and z-tests were used to assess significance in difference between rural and urban groups

### Linear regression analysis

In the univariate analysis, increasing rural proportion from entirely urban (rural proportion = 0) to entirely rural (rural proportion = 1) is associated with an increase in 0.70% point (95% CI: 0.49, 0.92) increase in mortality, adjusted for age, sex, and race and an increase in the percentage of eligible Medicare enrollees who receive an annual primary care visit (4.4% points; 95% CI: 2.4%, 6.5%). Increasing ADI was associated with a 0.02% point increase in age, sex, and race adjusted mortality (95% CI: 0.02%, 0.03%) and an increase in percentage of eligible Medicare enrollees who receive an annual primary care visit (0.14%; 95% CI: 0.11%, 0.17%). Rural proportion was negatively associated with price-adjusted Medicare reimbursement (-$970; 95% CI: -$1519, -$422) while ADI was positively associated with price-adjusted Medicare reimbursement ($32.13; 95% CI: $23.92, $40.36). Rural proportion and ADI were not significantly associated with percent of diabetic enrollees who receive hemoglobin A1c test nor percent of eligible female enrollees who receive mammograms (Table 2).

**Table 2:** Main effects table showing crude and adjusted regression coefficients for the association between percent rurality and the outcome measures. The adjusted model includes rural proportion and area deprivation index, a cumulative measure of income, education, employment, and housing quality in a given census block-group/neighborhood

	Rural proportion Crude (95% CI)	Area Deprivation Index Crude (95% CI)	Rural proportion Adjusted (95% CI)	Area Deprivation Index Adjusted (95% CI)
Total mortality ^1^	0.70%^†^ (0.49%, 0.92%)	0.02%^†^ (0.02%, 0.03%)	0.02% (-0.14%, 0.18%)	0.03%^†^ (0.02%, 0.03%)
Total Medicare reimbursement ^2^	-$2344.6^†^ (-$2847.9, -$1841.4)	-$20.23^†^ (-$29.23, -$11.34)	-$2209.5^†^ (-$2770.7, -$1648.4)	-$4.6 (-$13.6, $4.4)
Price-adjusted Medicare reimbursement ^2^	-$970.2^†^ (-$1518.5, -$421.9)	$32.13^†^ ($23.92, $40.36)	-$2272.8^†^ (-$2782.4, -$1763.2)	$48.2^†^ ($40.0, $56.4)
Annual primary care visit ^3^	4.4%^†^ (2.4%, 6.5%)	0.14%^†^ (0.11%, 0.17%)	0.8% (-1.3%, 2.9%)	0.13%^†^ (0.10%, 0.17%)
Diabetic enrollees who receive hemoglobin A1c test ^4^	1.02% (-0.57%, 2.62%)	-0.0005% (-0.03,0.03)	1.39% (-0.39%, 3.17%)	-0.01% (-0.04%, 0.02%)
Percent of eligible female enrollees who receive mammogram ^5^	-0.37% (-2.81%, 2.06%)	-0.04% (-0.08%,0.0008%)	0.93% (-1.77%, 3.62%)	-0.04%^†^ (-0.09%, -0.001%)

^1^Percent of deaths among Medicare enrollees adjusted for age, sex, and race

^2^Total annual reimbursements per Medicare enrollee ($, USD)

^3^Percent of Medicare enrollees who have at least 1 visit to a primary care clinician

^4^Average annual percent of diabetic Medicare enrollees age 65–75 who receive hemoglobin A1c test

^5^Average percent of female Medicare enrollees age 67–69 having at least one mammogram over a two-year period

^†^Denotes significance at the p < 0.05 level

When we included ADI in the multivariate linear regression model (Table 2), the association between rural proportion and mortality became non-significant and ADI had a significant, positive association with increased mortality (0.03%; 95% CI: 0.02%, 0.03%). Both ADI and proportion rurality were significantly associated with price-adjusted Medicare reimbursement, increasing ADI was associated with greater spending ($48.2; 95% CI: $40.0, $56.4) and increasing rural proportion was associated with less spending (-$2273; 95% CI: -$2782, -$1763).

## Discussion

Our study demonstrates that HRRs serving more rural populations experience a greater burden of socioeconomic disadvantage. The multivariate regression analysis shows that the one-year mortality rate among Medicare beneficiaries more strongly associated with socioeconomic deprivation within HRRs, rather than the rurality of the region. This suggests the increased mortality in rural areas is mediated by characteristics of the population and area: economic opportunity, education, employment, and housing, rather than the quality of care provided in the region for the HRR or the geographic characteristics that are inherent to rural living.

These findings are consistent with the conclusions of Long et al., 2018, who show that median income and percent in poverty are the primary drivers of disparities in rural mortality, rather than rural status, at the county level [3]. Our analysis adds to this previous work by incorporating where rural populations are receiving their healthcare, and we show that HRRs serving more rural populations have greater burden of sociodemographic deprivation which appears to be driving the adverse health outcomes in these more rural HRRs. Compared to their urban counterparts in 2018, 14.5% fewer rural residents hold bachelor’s degrees; rural individuals make, on average, $7,600 less per year; and there are 7.6% fewer adults in the labor force in rural areas [15, 16]. These data align with our findings of a positive correlation between rurality and ADI which we believe are contributing to disparities in rural mortality.

In *Deaths of Despair*, Case and Deaton (2020) discuss possible mechanisms by which income, education, and employment drive mortality disparities. They note education is protective against preventable diseases because information about disease prevention is more accessible to those with more education. Individuals age 25 and older with less than a Bachelor’s degree (BA) are four-times as likely to be current smokers and have higher rates of obesity (33% vs. 25%) compared those with a BA or more education [17]. Case and Deaton discuss the rise in “deaths of despair”, which refer to death caused by alcohol, drug-use, and suicide. Rates of deaths of despair are higher among those who are less educated compared to those with a 4-year degree, and in areas with higher rates of unemployment [17].

Additionally, we show that rural proportion is associated with lower overall healthcare utilization as measured by price-adjusted Medicare reimbursements and this relationship is not confounded by the level of sociodemographic disadvantage. This finding could be explained by higher rates of annual primary care visits in rural HRRs which leads to decreased downstream healthcare utilization and decreased overall Medicare spending [18, 19]. Our results suggest that more rural HRRs spend less per Medicare enrollee, and are able to achieve similar outcomes on select measures compared more urban HRRs, as shown by the lack of a significant difference in primary care quality and mortality by rurality when adjusted for ADI. This work contributes to a body of research in geographic variations in healthcare, pioneered by Wennberg and Fisher, that show that Medicare enrollees in higher-spending regions receive more care, but do not have better quality of– or access to– care, health outcomes, and satisfaction, compared to lower spending regions [18–21].

Further, Chandra and Skinner (2012) show that there are categories of hospital procedures that are costly and yield uncertain clinical value with variable benefits to patient quality of life [22]. These procedures, such as percutaneous coronary intervention (PCI), knee arthroscopy, and aggressive treatment for end-stage cancer, often require expensive infrastructure, technology, and specialized physicians, more commonly found in urban and academic medical centers. Colla et al. (2015) found that HRRs with higher per-capita spending had higher use of low-value services and greater ratios of specialists to primary care providers [23]. Rural clinics and hospitals experience lower patient volume, have fewer specialists, and have fewer expensive medical devices and technologies (such as robotic surgery, proton beam therapy), and are likely utilizing fewer low-value services, which could contribute to decreased spending and utilization in rural areas. One possible explanation for the lower utilization in more rural-serving HRRs with similar healthcare outcomes and quality compared to urban-serving HRRs is the discrepancy in technology and infrastructure between rural and urban hospitals. Further research is needed to investigate rates of expensive procedures, low-value procedures, and utilization patterns in HRRs serving rural vs. urban populations.

This study has several limitations, and the most important aspect is the single-year cross sectional analysis. Second, HRRs accurately localize care for approximately 88% of Medicare beneficiaries based on geographic locations, but that leaves a portion of beneficiaries who reside within HRR boundaries who do not receive their care at the given tertiary care center [24]. Third, we use one definition of rurality based on RUCA codes designated by HRSA. There may be other ways to measure rural populations at the zip code level which could yield different measures for proportion rurality in the areas served by HRRs [25]. Finally, we used ADI as a measure of sociodemographic deprivation, a composite measure of income, education, employment, and housing quality. As such, this analysis is not able to distinguish which factors are the primary drivers of the relationship between ADI and rurality and mortality. Further investigation is needed to see which ADI components are driving the association with rurality in order to impact rural disparities in mortality.

Disparities in rural mortality are driven by sociodemographic disadvantage in rural regions rather than the quality of healthcare available to rural populations. After adjusting for sociodemographic deprivation, we find that HRRs serving rural populations have comparable outcomes and primary care quality with less per capita Medicare spending.

## Electronic supplementary material

Below is the link to the electronic supplementary material.

Supplementary Material 1

## Data Availability

The datasets generated during and/or analyzed during the current study are available in the Dartmouth Atlas data repository, https://data.dartmouthatlas.org/.[11]

## Abbreviations

HRR: Hospital referral region
HSA: hospital service area
RUCA: rural-urban commuting area
ADI: area deprivation index
