# Supplementary material for: Rethinking rurality: using hospital referral regions to investigate rural-urban health outcomes
Source: BMC Health Serv Res. 2022 Nov 3;22:1312. doi: 10.1186/s12913-022-08649-0 (PMC9635085; doi:10.1186/s12913-022-08649-0)
Supplement: Supplementary file 1 — Supplementary Material 1 [file 12913_2022_8649_MOESM1_ESM.docx]

Supplemental Table 1. Mean values (95% CI) for middle quartiles as defined by proportion rural population.

|  | Quartile 1  *Most rural*  (N=77) | Quartile 2  (N = 77) | Quartile 3  (N = 75) | Quartile 4  *Most urban*  (N=77) |
| --- | --- | --- | --- | --- |
| Area Deprivation Index (ADI) | 61.7  (59.5, 64.0) | 60.0  (57.7, 62.2) | 52.9  (50.3, 55.6) | 41.6  (37.7, 45.4) |
| Total mortality^1^ | 4.50%  (4.40%, 4.59%) | 4.53%  (4.43%, 4.62%) | 4.27%  (4.19%, 4.36%) | 3.95%  (3.84%, 4.06%) |
| Total Medicare reimbursement^2^ | $9,506  ($9,324, $9,969) | $9,631  ($9,432, $9,829) | $10,099  ($9,878, $10,319) | $11,180  ($10,895, $11,466) |
| Price-adjusted Medicare reimbursement^2^ | $10,049  ($9,777, $10,322) | $10,226  ($9,962, $10,488) | $10,298  ($10,046, $10,549) | $10,694  ($10,427, $10,962) |
| Annual primary care visit^3^ | 80.8%  (79.9%, 81.7%) | 80.8%  (70.0%, 81.6%) | 80.2%  (79.3%, 81.0%) | 77.4%  (76.2%, 78.6%) |
| Diabetic enrollees who receive hemoglobin A1c test^4^ | 85.8%  (85.0%, 86.7%) | 86.1%  (85.2%, 87.0%) | 85.5%  (84.8%, 86.2%) | 85.6%  (85.0%, 86.2%) |
| Percent of eligible female enrollees who receive mammogram^5^ | 62.9%  (61.7%, 64.2%) | 62.9%  (61.8%, 64.0%) | 63.2%  (62.0%, 64.2%) | 64.5%  (63.0%, 65.4%) |

Quartile 1 is defined by rural proportion ≥ 0.38, quartile 2 by rural proportion < 0.38 & ≥ 0.20, quartile 3 by rural proportion <0.20 & > 0.04, and quartile 4 by rural proportion ≤ 0.04.

^1^Percent of deaths among Medicare enrollees adjusted for age, sex, and race

^2^Total annual reimbursements per Medicare enrollee (parts A & B), ($, USD)

^3^Percent of Medicare enrollees who have at least 1 visit to a primary care clinician

^4^Average annual percent of diabetic Medicare enrollees age 65-75 who receive hemoglobin A1c test

^5^Average percent of female Medicare enrollees age 67-69 having at least one mammogram over a two-year period

**^*^**T-tests and z-tests were used to assess significance in difference between rural and urban groups.
